# Immunogenetic Profiling of SLE and LN among Jordanian Patients

**DOI:** 10.3390/jpm12121955

**Published:** 2022-11-25

**Authors:** Sawsan I. Khdair, Rawan Al-Bdour, Wassan Jarrar, Alaa Hammad, Aya Al-Jayeh, Mohammad Masa’deh, Marwan Adwan, Randa Farah

**Affiliations:** 1 Department of Pharmacy, Faculty of Pharmacy, Al-Zaytoonah University of Jordan, Amman 11733, Jordan; 2 Department of Medicine, The University of Jordan, Amman 11942, Jordan

**Keywords:** HLA class II genes, systemic lupus erythematosus, lupus nephritis, Jordan

## Abstract

Systemic Lupus Erythematosus (SLE) is a prolonged inflammatory autoimmune disease, which is characterized by a high titer of serological autoantibodies. Interactions between environmental and genetic factors play a crucial role in the pathogenesis of SLE. Human Leukocyte Antigen (HLA) genes, namely HLA-class II genes, are one of the main candidate genes that increase susceptibility to SLE. The aim of this study was to investigate, for the first time, the association of HLA-DRB1 and HLA-DQB1 genes among Jordanian patients diagnosed with SLE and Lupus Nephritis (LN) using the Polymerase Chain Reaction-Sequence-Specific Primer (PCR-SSP) technique. This study showed that SLE is positively associated with DRB1*0301, DRB1*1101, DRB1*1102 and HLA-DQB1*0601. Furthermore, HLA-DRB1*0301, DRB1*1101, HLA-DRB1*1501 and HLA-DQB1*0601 were found to be linked to SLE patients with LN. In addition, haplotypes HLA-DRB1*0301/DQB1*0201 and HLA-DRB1*1501/DQB1*0601 were found to be linked to SLE and LN. Our findings may serve as possible predictive markers for early screening for LN risk in SLE patients. In light of these results, the role of HLA gene polymorphisms may help in understanding the clinical course, prognosis of the disease and developing better treatment strategies for SLE patients. In addition, it may help in early diagnosis, prevention, intervention and management of the disease.

## 1. Introduction

Systemic Lupus Erythematosus (SLE) is a long-lasting inflammatory autoimmune disease that frequently affects females. SLE is characterized by the development of autoreactive B and T cells against self-antigens through the formation of antinuclear antibodies, anti-double stranded DNA antibodies and immune complexes [1]. The clinical picture of the disease varies from modest rashes or joints disease to organ damage and life-threatening complications such as glomerulonephritis and hematologic diseases. Importantly, it is unpredictable which complications will affect a particular patient [2]. Furthermore, there is a remarkable difference in the incidence as well as the prevalence of SLE disease in different ethnicities. The total global incidence and prevalence of SLE varies from 1.5 to 11 per 100,000 individuals, and between 13 to 7713.5 per 100,000 persons, respectively [3]. Although the etiology or the underlying pathophysiological mechanism that stimulates the autoimmune response in SLE patients, like many other autoimmune diseases, is still unknown, several factors including genetic, environmental, hormonal, epigenetic and immune-regulatory factors play a role in SLE susceptibility [4]. Over the years, genome-wide association studies (GWAS), have importantly enriched our knowledge and understanding of the genetic basis of SLE [5]. So far, GWAS and other independent studies mainly in European and Asian populations, have revealed around 100 genetic loci that may increase susceptibility to SLE, which emphasizes the essential role of genetic factors in predisposition to SLE [6,7]. Human leukocyte antigens (HLA), specifically HLA-class II genes, are one of the main candidate genes that increase the susceptibility to SLE [8,9]. Yet, it is unclear exactly how HLA predisposes an individual to AID. Molecular mimicry is a suggested hypothesis in which a foreign immunogen has structural or sequence that matches with self-antigens. Amino acids with similar chemical characteristics are able to bind at the MHC peptide binding cleft, which can be recognized by T-cell receptor (TCR). However, the peptide sequence binding to MHC class II molecules and forming MHC/peptide complex which is presented to T cells were found to be dependent on amino acid properties [10]. Recent studies suggested that amino acid alterations in some HLA genes were the bases of major SLE association in different populations. The amino acid locations 11, 13, 26 and 37 on the epitope-binding cleft of HLA-DRB1, the amino acid location 37 of the HLA-DQB1 and the locations 70 and 9 in HLA-A and HLA-B, respectively, were found to be responsible for HLA-SLE association [11,12].

Several studies among different populations had attempted to deduce the HLA-SLE association to identify the possible HLA genes that may increase the risk to SLE. For example, in studies performed on European SLE patients, high risk of SLE was linked to HLA-B*0801, HLA-DRB1*0301 and HLA-DRB1*0801 [13]. In addition, HLA class I alleles B*0801 and B*1801 and class II alleles DQB1*0201, DRB3*02 and DQA*0102 were reported to be associated with SLE in European ancestry SLE patients [14]. On the other hand, alleles with increased susceptibility to SLE in Asian patients were HLA-DRB1*0901, HLA-DRB1*1501 and HLA-DQB1*0602 [15,16,17]. Furthermore, a study that was conducted in the Tunisian population found that HLA-DRB1*03 was associated with SLE [18], while HLA-DQB1*06 has been linked to SLE in Egyptians [19]. In addition, DRB1*0301/DQA1*0501/DQB1*0201 and DRB1*1501/03/DQA1*0102/DQB1*0601 haplotypes were considered SLE-risk alleles in European, African and Hispanic American ancestries [20]. To date, there is no study that investigates whether there is an association between HLA genes and SLE among Jordanians. The main goal of this study was to investigate HLA-DRB1 and HLA-DQB1 polymorphisms in Jordanian SLE patients in comparison to a control healthy group. In addition, another goal of the study was to figure out the HLA alleles/haplotypes that are linked to lupus nephritis (LN) among Jordanian SLE patients. Identifying the genes associated with SLE among Jordanian patients could help in identifying individuals at higher risk of developing the disease.

## 2. Materials and Methods

### 2.1. Subject Selection

A total of 74 healthy controls and 80 SLE patients took part in this study. The patients were enrolled from the department of rheumatology at the Jordan University Hospital (JUH) in Amman, Jordan from March 2021 to January 2022. SLE patients were diagnosed according to the guidelines of the American College of Rheumatology (ACR) [21]. An inclusion criterion of this study was that all participants of the study should not be relatives and should be of Jordanian origin. In addition, an exclusion criterion for patients was having other autoimmune diseases other than SLE. Subjects in the control group having autoimmune diseases in their family history were also excluded. The inclusion and exclusion criteria for the control group were based on a questionnaire used for this study. Patients’ demographic and clinical information were gained from hospital records. Institutional review board (IRB) approval number (146/2021) was gained from the ethical committee at the JUH and all procedures were performed in accordance with the ethical standards of the institution and the national research committee in addition to the principles of the declaration of Helsinki. An informed consent was obtained from all participants. Patient and control volunteers were not involved directly in the design and conduct of this study. However, they were completely informed about the procedure, the aim and the possible outcomes of this study. In addition, the importance of this study as well as the value of enriching the scientific society with genetical background of SLE patients was explained to the volunteers.

### 2.2. DNA Extraction and HLA Genotyping

Three milliliters of peripheral whole blood was used for DNA extraction by using Wizard^®^ Genomic DNA purification kit as described previously [22,23]. DNA has been quantified using the NanoDrop spectrophotometer (Quawell DNA/Protein Analyzer, Montreal, QC, Canada). DNA purity (A260/A280) was between 1.7 and 1.8 and the average concentration of the DNA samples was about 50 ng. DNA samples used were stored at −20 °C until use. HLA-DRB1 and HLA-DQB1 genotyping was performed by the polymerase chain reaction-sequence-specific primers method (PCR-SSP) using the micro SSP kit of One Lambda (Roscoe Blvd, West Hills, ON, Canada) conferring to the manufacturer’s protocol.

### 2.3. Statistical Analysis

The Statistical Package for Social Sciences (SPSS) software (version 26) was used for statistical analysis (IBM analytics, Armonk, NY, USA). Differences in the frequencies (%) of HLA-DRB1 and HLA-DQB1 alleles and haplotypes of SLE patients’ group and a healthy group were analyzed using Chi-square test. Odds ratio (OR) was calculated with a 95% Confidence Interval (CI). In case the frequency of any of the studied groups was equal to zero, Haldane–Anscombe correction [24,25] was applied to correct the bias using the MedCalc software Ltd. (Ostend, Belgium) [26,27] for measuring the *p*-value, OR and 95% CI as described previously [28,29]. Differences were considered significant when the *p* value was less than 0.05.

## 3. Results

### 3.1. Demographic Data of the Participants

In total, 154 participants took part in this study (74 healthy controls and 80 SLE patients). The average age (mean ± SD) of the control group and SLE patients was 29.10 ± 7.02 and 38.91 ± 12.46 years, respectively. The control group included 45 (60.8%) males and 29 (39.2%) females, while the SLE patients’ group consisted of 7 (8.8%) males and 73 (91.3%) females. Furthermore, the mean age of SLE disease onset was 27.80 ± 11.08 years and the mean duration of the disease was 11.11 ± 8.57 years. In addition, almost half of the SLE patients 39 (48.8%) suffered from LN.

### 3.2. HLA-DRB1 Allele Frequencies among SLE Patients and Healthy Controls

Table 1 shows the distribution of HLA-DRB1 allele frequencies in SLE patients and controls. HLA-DRB1*0301 (*p* = 0.005, OR=2.60), HLA-DRB1*1101 (*p* = 0.041, OR = 1.86) and HLA-DRB1*1102 (*p* = 0.02, OR = 2.64) showed a positive association with SLE, with a higher frequency of the alleles in the SLE patients’ group in comparison to the control group. On the other hand, HLA-DRB1*0101 (*p* = 0.022, OR = 0.20), HLA-DRB1*0403 (*p* = 0.023, OR = 0.04), HLA-DRB1*0701 *(p* = 0.004, OR = 0.35), HLA-DRB1*1301 (*p* = 0.016, OR = 0.27) and DRB1*1401 (*p* = 0.042, OR = 0.05) were negatively linked to SLE.

### 3.3. HLA-DQB1 Allele Frequencies among SLE Patients and Healthy Controls

HLA-DQB1 alleles frequencies are presented in Table 2. The HLA-DQB1 allele that showed a statistically significant positive association with SLE was DQB1*0601 (*p* ≤ 0.001, OR = 11.03), with a higher frequency of the alleles in the SLE patients’ group in comparison to the control group. However, HLA-DQB1*0603 (*p* = 0.028, OR = 0.04) is suggested to be a protective allele since it was totally absent in the SLE patients’ group.

### 3.4. HLA-DRB1/DQB1 Haplotypes among SLE Patients and Healthy Controls

Table 3 shows a large variance in the putative HLA-DRB1/DQB1 haplotypes among our groups (54 haplotypes) with an increase in the frequency of DRB1*0301/DQB1*0201 (*p* = 0.037, OR = 2.20) and DRB1*1501/DQB1*0601 (*p* < 0.001, OR = 9.84) haplotypes in SLE patients in comparison to controls. On the other hand, the following HLA-DRB1/DQB1 putative haplotypes are suggested to be protective haplotypes since they were totally absent in the SLE patients: DRB1*0403/DQB1*0302 (*p* = 0.034, OR = 0.05), DRB1*0701/DQB1*0201 (*p* = 0.001, OR = 0.24), DRB1*1301/DQB1*0603(*p* = 0.028, OR = 0.04) and DRB1*1501-DQB1*0602 (*p* = 0.028, OR = 0.04).

### 3.5. HLA-DRB1 Allele Frequencies among SLE Patients with LN and Healthy Controls

Table 4 shows the HLA-DRB1 allele frequency in SLE patients with LN and healthy control groups. The results showed that the HLA-DRB1*0301 (*p* = 0.006, OR = 2.89), DRB1*1101 (*p* = 0.040, OR = 2.06) and DRB1*1501 (*p* = 0.043, OR = 2.27) alleles show a positive association with the SLE with LN group, with a higher allele frequency in the LN group in comparison to the control group. In contrast, DRB1*0701 (*p* = 0.040, OR = 0.39) and DRB1*1301 (*p* = 0.026, OR = 0.14) may be suggested as protective alleles, as they show a significant negative association to SLE with LN.

### 3.6. HLA-DQB1 Allele Frequencies among SLE Patients with LN and Healthy Controls

Table 5 shows the HLA-DQB1 allele frequency in the SLE with LN group and the control group. The results revealed that the HLA-DQB1*0601 allele was positively linked to LN (*p* ≤ 0.001, OR = 18.84), while the DQB1*0602 (*p* = 0.044, OR = 0.06) allele was negatively linked to LN susceptibility.

### 3.7. HLA-DRB1/DQB1 Haplotype Frequencies among SLE Patients with LN and Healthy Controls

Table 6 shows the HLA-DRB1/DQB1 haplotype frequencies in SLE with LN and the control groups. Our results revealed an increase in the haplotype frequency of DRB1*0301/DQB1*0201 (*p* = 0.032, OR = 2.50) and DRB1*1501-DQB1*0601 (*p* < 0.001, OR = 15.97) in LN patients in comparison to the control group. In contrast, the DRB1*0701-DQB1*0201 (*p* = 0.016, OR = 0.28) haplotype was suggested protective against LN development.

### 3.8. HLA-DRB1 and -DQB1 Allele and Haplotype Frequencies among SLE Patients without LN and SLE with LN

Our findings showed that the allele frequencies of HLA-DRB1*0301 and HLA-DRB1*1101 were high in both groups, SLE patients with LN and SLE patients without LN without significant differences (Table S1). However, results showed that the HLA-DRB1*1501 and DQB1*0601 alleles were significantly increased (*p* = 0.042 and *p* = 0.007, respectively) among SLE patients with LN in comparison to SLE patients without LN (Table S1). In addition, our findings revealed that the HLA-DRB1*1501/DQB1*0601 haplotype is significantly increased (*p* = 0.021) among SLE patients with LN in comparison to SLE patients without LN (Table S2).

## 4. Discussion

Systemic Lupus Erythematosus (SLE) is a prolonged autoimmune disease, with a wide array of manifestations that may affect any organ. Renal manifestations were estimated to reach 50.4% among Arab SLE patients and 47.62% in Jordanian SLE patients [30]. This study investigates for the first time the association of HLA-DRB1 and HLA-DQB1 polymorphisms in Jordanian SLE patients as well as SLE with LN patients in comparison to control healthy individuals.

In Jordanian SLE patients, our results revealed a positive association of DRB1*0301 as it seems to increase the susceptibility to SLE disease in the Jordanian population. Comparable results were also observed in studies done on SLE patients in Caucasian US [31], Latin American [32], Tunisian [18,33], Taiwanese [34], Italian [35], Portuguese [36], British and European [13,37], Pakistani [38] and Korean populations [11]. Furthermore, both DRB1*1101 and DRB1*1102 which have shown a significant association with SLE susceptibility in Jordanian patients in our study, were also shown to be associated with SLE predisposition in the Pakistani [38] as well as the Iranian and west Indian population [39,40]. Nevertheless, the DRB1*1101 allele was considered a protective allele in Latin Americans [32]. Regarding the HLA-DQB1 alleles, our results reported a positive association of HLA-DQB1*0601 allele with SLE disease susceptibility. This finding is consistent with previous studies that was conducted on Iranian [41] and on Hungarian SLE patients [42]. Furthermore, our analysis showed that the following HLA-DRB1 alleles were negatively linked to SLE susceptibility: *0101, *0403, *0701, *1301 and *1401 and may thus be considered protective alleles against SLE development. Notably, DRB1*0403, DRB1*1401 and DQB1*0603 can be considered protective alleles against SLE development since they were totally absent in the Jordanian SLE patients’ group.

LN is one of the most severe complications of SLE disease and is a crucial driver of mortality and morbidity in SLE [3]. Regarding Jordanian SLE patients with LN, our data demonstrated significant association between the HLA-DRB1*0301 allele and LN susceptibility when compared to healthy controls. This positive association was also found in Swedish [43], as well as Italian LN patients [35]. In addition, our findings revealed a positive link of the HLA-DRB1*1501 allele with LN in comparison to the healthy controls, and this result is aligned with the findings of studies done on SLE patients with LN in other populations such as Indonesian [44], Moroccan [45], Saudi [46] as well as Brazilian populations [47]. Furthermore, in line with our results, a meta-analysis study in 2014 stated that HLA-DR3 (DRB1*0301) and DR2 (DRB1*1501) increased the risk for LN development [48,49]. Nevertheless, while our results revealed a positive association of HLA-DR11 (DRB1*1101) allele with LN in Jordanian patients when compared to healthy controls, DR11 alleles was reported to be a protective factor against LN development as stated by a meta-analysis study and other independent studies [44,46,47,48,49]. This remarkable difference in literature concerning protective alleles of SLE is in part due to genetic differences among populations and environmental exposures. Regarding the HLA-DQB1 alleles, our results reported a positive association of HLA-DQB1*0601 allele in SLE with LN. This finding is consistent with what has been reported in Iranians [41]. In addition, the HLA-DQB1*0602 allele can be considered a protective allele against LN development since the allele was totally absent in the Jordanian SLE with LN patients’ group. Furthermore, intra-group analysis between the frequency of alleles in SLE patients without LN and SLE patients with LN showed that HLA-DRB1*0301 and HLA-DRB1*1101 were high in both groups with no statistically significant differences. Nevertheless, comparison between the frequency of alleles in SLE patients without LN and SLE patients with LN confirms our conclusions about the role of HLA-DRB1*1501 and DQB1*0601 alleles in increasing susceptibility of LN in SLE patients.

Furthermore, two haplotypes, namely HLA-DRB1*0301/DQB1*0201 and HLA-DRB1*1501/DQB1*0601, have been linked to SLE Jordanian patients and LN in comparison to healthy controls. Our results suggested that the HLA-DRB1*1501-DQB1*0601 haplotype is a risk factor for SLE and LN in the Jordanian SLE patients, whereas DQB1*0602 allele suppressed the immune response to SLE predisposition of DRB1*1501 as the haplotype HLA-DRB1*1501-DQB1*0602 is suggested to be a protective haplotype in the Jordanians. Nevertheless, a study performed in the Italian population showed that DRB1*1501 significantly increased the likelihood of developing LN in combination with the DQA1*0101 allele (HLA-DRB1*1501-DQA1*0101), while DQA1*0102 significantly reduced the nephritogenic effects of DRB1*1501 (HLA-DRB1*1501-DQA1*0102) in Italian SLE patients [35]. Moreover, the following haplotypes: DRB1*0403/DQB1*0302, DRB1*0701/DQB1*0201 and DRB1*1301/DQB1*0603 may be considered protective haplotypes since they were negatively linked to SLE patients. In addition, intra-group analysis between the frequency of haplotypes in SLE patients without LN and SLE patients with LN confirmed that the HLA-DRB1*1501/DQB1*0601 haplotype is linked to LN in SLE patients in Jordan. All in all, varying ethnic genetic backgrounds of the different investigated populations may be the cause of the disparity between our findings and those of other populations and the role of HLA class II alleles association or protection with SLE disease is still debatable among different populations.

In summary, our results revealed a significant association of the following HLA-DRB1 alleles: *0301, *1101, *1102, and the HLA-DQB1*0601 allele with SLE in Jordanian patients. Furthermore, DRB1*0301, DRB1*1501 and HLA-DQB1*0601 were found to be significantly associated with LN development in SLE patients. Previous studies which indicated that the class II alleles HLA-DR2 (DRB1*1501) and HLA-DR3 (DRB1*0301) confer a two- to three-fold greater risk for SLE development is in agreement with our findings [11,31,32,33,34,35,36,37,46,47,50]. In addition, two putative haplotypes, HLA-DR3 (HLA-DRB1*0301/-DQB1*0201) and HLA-DR2 (HLA-DRB1*1501/DQB1*0601), were found to be linked to SLE Jordanian patients and LN when compared to healthy controls, while DRB1*0403/DQB1*0302, DRB1*0701/DQB1*0201 and DRB1*1301/DQB1*0603 may be considered protective haplotypes. In addition, HLA-DRB1*11 allele was reported in our study to be associated with Jordanian SLE and LN patients in comparison with healthy population which is infrequently found to be implicated in SLE or LN. These results could provide useful information for diagnosis and the determination of prognosis in Jordanian SLE patients. This variation in the results may be attributed to genetic ethnic diversity among different populations and environmental exposures. We acknowledge that the sample size of this study was limited. However, all SLE patients that visited the department of rheumatology at JUH during the period of the recruitment process were included in this study.

## 5. Conclusions

In conclusion, analysis of the immunogenetic profile of SLE and LN Jordanian patients unveiled several alleles that could be considered risk factors for SLE disease development, namely DRB1*0301, *1101, *1501 and DQB1*0601. Furthermore, DRB1*0301, DRB1*1501 and HLA-DQB1*0601 appear to increase the risk factor for LN in Jordanian SLE patients. Additionally, haplotypes HLA-DR3 and HLA-DR2 were found to be linked to SLE Jordanian patients and LN, while DRB1*0403/DQB1*0302, DRB1*0701/DQB1*0201 and DRB1*1301/DQB1*0603 are protective haplotypes against SLE development. Therefore, these alleles/haplotypes may serve as possible predictive markers for early screening for SLE disease as well as screening for LN risk in SLE patients. Early detection and timely diagnosis of LN are crucial as LN is a leading cause of morbidity and mortality in SLE patients. This would make it easier for SLE patients who may be at risk of getting LN to take the necessary precautions to delay the progression of the disease. In addition, knowledge of the causative genes will reduce the progression of the disease with proper treatment according to the patient’s genetic make-up. Moreover, knowing how these alleles function may aid in creating more effective treatment plans for SLE and its associated complications such as LN. For the diagnosis, prevention, intervention and management of these disorders, it is also crucial to comprehend the etiology and progression of SLE and LN and to identify all the potential risk genes that might influence the pathogenesis of LN. Similar to several other autoimmune diseases, SLE remains a clinical mystery for physicians and patients, and for this reason, a multidisciplinary management approach is often needed for SLE patients, so in the future, widespread genome and clinical studies are suggested.

## Figures and Tables

**Table 1:** Frequencies of HLA-DRB1 alleles in SLE patients versus healthy controls in Jordanians.

	Control		SLE				
Allele	*n*= 148	(%)	*n* = 160	(%)	*p* Value	OR	95% CI
DRB1*0101	**9**	**6.1**	**2**	**1.3**	**0.022**	**0.20**	**0.04–0.92**
DRB1*0103	2	1.4	0	0	0.274	0.18	0.01–3.83
DRB1*0301	**13**	**8.8**	**32**	**20.0**	**0.005**	**2.60**	**1.30–5.17**
DRB1*0302	0	0	3	1.9	0.213	6.60	0.34–128.9
DRB1*0401	0	0	5	3.1	0.112	10.51	0.58–191.7
DRB1*0402	3	2.0	1	0.6	0.278	0.31	0.03–2.96
DRB1*0403	**11**	**7.4**	**0**	**0**	**0.023**	**0.04**	**0.002–0.64**
DRB1*0405	0	0	1	0.6	0.530	2.79	0.11–69.10
DRB1*0701	**26**	**17.6**	**11**	**6.9**	**0.004**	**0.35**	**0.17–0.73**
DRB1*0801	3	2.0	0	0	0.178	0.13	0.01–2.53
DRB1*1001	8	5.4	13	8.1	0.344	1.55	0.62–3.85
DRB1*1101	**20**	**13.5**	**36**	**22.5**	**0.041**	**1.86**	**1.02–3.39**
DRB1*1102	**8**	**5.4**	**21**	**13.1**	**0.020**	**2.64**	**1.13–6.17**
DRB1*1103	0	0	1	0.6	0.530	2.79	0.11–69.10
DRB1*1201	1	0.7	4	2.5	0.206	3.77	0.42–34.16
DRB1*1301	**13**	**8.8**	**4**	**2.5**	**0.016**	**0.27**	**0.09–0.84**
DRB1*1302	0	0	1	0.6	0.530	2.79	0.11–69.10
DRB1*1303	7	4.7	2	1.3	0.070	0.26	0.05–1.25
DRB1*1401	**8**	**5.4**	**0**	**0**	**0.0** **42**	**0.05**	**0.003–0.90**
DRB1*1402	0	0	2	1.3	0.320	4.69	0.22–98.39
DRB1*1404	1	0.7	0	0	0.470	0.31	0.01–7.58
DRB1*1501	13	8.8	20	12.5	0.292	1.48	0.71–3.10
DRB1*1502	1	0.7	0	0	0.470	0.31	0.01–7.58
DRB1*1601	1	0.7	1	0.6	0.956	0.93	0.06–14.92

SLE: Systemic Lupus Erythematosus; *n*: number of individuals; (%): allele frequency; OR: odds ratio; CI: confidence interval; *p*: significance level; (*p* value < 0.05).

**Table 2:** Frequency of HLA-DQB1 alleles in SLE patients versus healthy controls in Jordanians.

	Control		SLE				
Allele	*n* = 148	(%)	*n* = 160	(%)	*p* Value	OR	95% CI
DQB1*0201	33	22.3	35	21.9	0.929	0.98	0.57–1.67
DQB1*0202	2	1.4	0	0	0.274	0.18	0.01–3.83
DQB1*0301	38	25.7	55	34.4	0.097	1.52	0.93–2.48
DQB1*0302	17	11.5	18	11.3	0.948	0.98	0.48–1.98
DQB1*0303	3	2.0	2	1.3	0.590	0.61	0.10–3.71
DQB1*0401	0	0	2	1.3	0.320	4.69	0.22–98.39
DQB1*0402	2	1.4	2	1.3	0.937	0.92	0.13–6.65
DQB1*0501	24	16.2	17	10.6	0.149	0.61	0.32–1.20
DQB1*0502	1	0.7	1	0.6	0.956	0.93	0.06–14.92
DQB1*0503	1	0.7	0	0	0.470	0.31	0.01–7.58
DQB1*0601	**2**	**1.4**	**21**	**13.1**	**<0.001**	**11.03**	**2.54–47.91**
DQB1*0602	15	10.1	7	4.4	0.050	0.41	0.16–1.03
DQB1*0603	**10**	**6.8**	**0**	**0**	**0.028**	**0.04**	**0.002–0.71**

SLE: Systemic Lupus Erythematosus; *n*: number of individuals; (%): allele frequency; OR: odds ratio; CI: confidence interval; *p*: significance level (*p* value < 0.05).

**Table 3:** The frequency of HLA-DRB1/DQB1 haplotypes among SLE and healthy controls in Jordanians.

	Controls		SLE				
Haplotype	*n* = 148	(%)	*n* = 160	(%)	*p* Value	OR	95% CI
0101/0201	0	0	2	1.3	0.320	4.69	0.22–98.39
0101/0301	2	1.4	0	0.0	0.274	0.18	0.01–3.83
0101/0501	7	4.7	0	0.0	0.053	0.06	0.003–1.04
0103/0301	2	1.4	0	0.0	0.274	0.18	0.01–3.83
**0301/0201**	**11**	**7.4**	**24**	**15.0**	**0.037**	**2.20**	**1.04–4.66**
0301/0301	0	0.0	1	0.6	0.530	2.79	0.11–69.10
0301/0302	0	0.0	4	2.5	0.151	8.54	0.46–159.9
0301/0303	1	0.7	1	0.6	0.956	0.93	0.06–14.92
0301/0501	1	0.7	1	0.6	0.956	0.93	0.06–14.92
0301/0602	0	0.0	1	0.6	0.530	2.79	0.11–69.10
0302/0402	0	0.0	3	1.9	0.213	6.60	0.34–128.9
0401/0301	0	0.0	2	1.3	0.320	4.69	0.22–98.39
0401/0302	0	0.0	3	1.9	0.213	6.60	0.34–128.9
0402/0302	3	2.0	1	0.6	0.278	0.30	0.03–2.96
**0403/0302**	**9**	**6.1**	**0**	**0.0**	**0.034**	**0.05**	**0.003–0.79**
0403/0402	2	1.4	0	0.0	0.274	0.18	0.01–3.83
0405/0401	0	0.0	1	0.6	0.530	2.79	0.11–69.10
**0701/0201**	**24**	**16.2**	**7**	**4.4**	**0.001**	**0.24**	**0.10–0.57**
0701/0301	0	0.0	2	1.3	0.320	4.69	0.22–98.39
0701/0303	2	1.4	1	0.6	0.517	0.46	0.04–5.12
0702/0201	0	0.0	1	0.6	0.530	2.79	0.11–69.10
0801/0301	3	2.0	0	0.0	0.178	0.13	0.01–2.53
1001/0201	0	0.0	1	0.6	0.530	2.79	0.11–69.10
1001/0302	2	1.4	1	0.6	0.517	0.46	0.04–5.12
1001/0501	6	4.1	10	6.3	0.386	1.58	0.56–4.45
1001/0602	0	0.0	1	0.6	0.530	2.79	0.11–69.10
1101/0301	18	12.2	26	16.3	0.306	1.40	0.73–2.68
1101/0302	1	0.7	5	3.1	0.120	4.74	0.55–41.07
1101/0501	0	0.0	4	2.5	0.151	8.54	0.46–159.9
1101/0602	1	0.7	0	0.0	0.470	0.31	0.01–7.58
1102/0301	7	4.7	17	10.6	0.054	2.40	0.96–5.95
1102/0302	0	0.0	1	0.6	0.530	2.79	0.11–69.10
1102/0601	0	0.0	2	1.3	0.320	4.69	0.22–98.39
1102/0602	1	0.7	2	1.3	0.608	1.86	0.17–20.74
1103/0201	0	0.0	1	0.6	0.530	2.79	0.11–69.10
1201/0301	1	0.7	4	2.5	0.206	3.77	0.42–34.12
1301/0301	0	0.0	1	0.6	0.530	2.79	0.11–69.10
1301/0302	0	0.0	1	0.6	0.530	2.79	0.11–69.10
1301/0501	3	2.0	0	0.0	0.178	0.13	0.01–2.53
1301/0602	0	0.0	2	1.3	0.320	4.69	0.22–98.39
**1301/0603**	**10**	**6.8**	**0**	**0.0**	**0.028**	**0.04**	**0.002–0.71**
1302/0302	0	0.0	1	0.6	0.530	2.79	0.11–69.10
1303/0201	0	0.0	1	0.6	0.530	2.79	0.11–69.10
1303/0301	5	3.4	0	0.0	0.090	0.08	0.01–1.48
1303/0302	2	1.4	1	0.6	0.517	0.46	0.04–5.12
1401/0501	5	3.4	0	0.0	0.090	0.08	0.01–1.48
1401/0602	3	2.0	0	0.0	0.178	0.13	0.01–2.53
1402/0301	0	0.0	2	1.3	0.320	4.69	0.22–98.38
1404/0503	1	0.7	0	0.0	0.470	0.31	0.01–7.58
1501/0201	0	0.0	1	0.6	0.530	2.79	0.11–69.10
1501/0501	1	0.7	0	0.0	0.470	0.31	0.01–7.58
**1501/0601**	**2**	**1.4**	**19**	**11.9**	**<0.001**	**9.84**	**2.25–43.01**
**1501/0602**	**10**	**6.8**	**0**	**0.0**	**0.028**	**0.04**	**0.002–0.71**
1502/0501	1	0.7	0	0.0	0.470	0.31	0.01–7.58
1601/0502	1	0.7	1	0.6	0.956	0.93	0.06–14.92

SLE: Systemic Lupus Erythematosus; *n*: number of individuals; (%): allele frequency; OR: odds ratio; CI: confidence interval; *p*: significance level (*p* value < 0.05).

**Table 4:** HLA-DRB1 allele frequencies of SLE with LN and healthy controls.

	Control		SLE with Nephritis				
Allele	*n* = 148	(%)	*n* = 78	(%)	*p* Value	OR	95% CI
DRB1*0101	9	6.1	0	0	0.104	0.09	0.01–1.63
DRB1*0103	2	1.4	0	0	0.526	0.37	0.02–7.87
DRB1*0301	**13**	**8.8**	**17**	**21.8**	**0.006**	**2.89**	**1.32–6.33**
DRB1*0401	0	0	3	3.8	0.084	13.77	0.70–270
DRB1*0402	3	2.0	1	1.3	0.686	0.63	0.06-6.14
DRB1*0403	11	7.4	0	0	0.076	0.08	0.004–1.31
DRB1*0701	**26**	**17.6**	**6**	**7.7**	**0.040**	**0.39**	**0.15–1.00**
DRB1*0801	3	2.0	0	0	0.382	0.26	0.01–5.19
DRB1*1001	8	5.4	4	5.1	0.930	0.95	0.28–3.25
DRB1*1101	**20**	**13.5**	**19**	**24.4**	**0.040**	**2.06**	**1.02–4.15**
DRB1*1102	8	5.4	8	10.3	0.176	2.00	0.72–5.55
DRB1*1103	0	0	1	1.3	0.286	5.75	0.23–142.8
DRB1*1201	1	0.7	2	2.6	0.238	3.87	0.35–43.35
DRB1*1301	**13**	**8.8**	**1**	**1.3**	**0.026**	**0.14**	**0.02–1.05**
DRB1*1302	0	0	1	1.3	0.286	5.75	0.23–142.8
DRB1*1303	7	4.7	1	1.3	0.182	0.26	0.03–2.16
DRB1*1401	8	5.4	0	0	0.124	0.11	0.01–1.85
DRB1*1404	1	0.7	0	0	0.775	0.63	0.03–15.56
DRB1*1501	**13**	**8.8**	**14**	**17.9**	**0.043**	**2.27**	**1.01–5.11**
DRB1*1502	1	0.7	0	0	0.775	0.63	0.03–15.56
DRB1*1601	1	0.7	0	0	0.775	0.63	0.03–15.56

SLE: Systemic Lupus Erythematosus; *n*: number of individuals; (%): allele frequency; OR: odds ratio; CI: confidence interval; *p*: significance level (*p* value < 0.05).

**Table 5:** HLA-DQB1 allele frequencies in SLE with lupus nephritis (LN) and healthy controls.

	Control		SLE with Nephritis				
Allele	*n* = 148	(%)	*n* = 78	(%)	*ρ* Value	OR	95% CI
DQB1*0201	33	22.3	21	26.9	0.438	1.28	0.68–2.42
DQB1*0202	2	1.4	0	0	0.526	0.37	0.02–7.87
DQB1*0301	38	25.7	26	33.3	0.224	1.45	0.80–2.63
DQB1*0302	17	11.5	8	10.3	0.779	0.88	0.36–2.14
DQB1*0303	3	2.0	1	1.3	0.686	0.63	0.06–6.14
DQB1*0402	2	1.4	0	0	0.526	0.37	0.02–7.87
DQB1*0501	24	16.2	6	7.7	0.073	0.43	0.17–1.10
DQB1*0502	1	0.7	0	0	0.775	0.63	0.03–15.56
DQB1*0503	1	0.7	0	0	0.775	0.63	0.03–15.56
DQB1*0601	**2**	**1.4**	**16**	**20.5**	**<0.001**	**18.84**	**4.21–84.40**
DQB1*0602	**15**	**10.1**	**0**	**0**	**0.044**	**0.06**	**0.003–0.93**
DQB1*0603	10	6.8	0	0	0.089	0.08	0.005–1.45

SLE: Systemic Lupus Erythematosus; *n*: number of individuals; (%): allele frequency; OR: odds ratio; CI: confidence interval; *p*: significance level (*p* value < 0.05).

**Table 6:** The frequency of DRB1*/DQB1* haplotypes among controls and Lupus Nephritis groups in the Jordanian population.

	Controls		SLE with Nephritis		-		
Haplotype	*n* = 148	(%)	*n* =78	(%)	*p* Value	OR	95% CI
0101/0301	2	1.4	0	0	0.526	0.37	0.02–7.87
0101/0501	7	4.7	0	0	0.149	0.12	0.01–2.13
0103/0301	2	1.4	0	0	0.526	0.37	0.02–7.87
**0301/0201**	**11**	**7.4**	**13**	**16.7**	**0.032**	**2.50**	**1.06–5.86**
0301/0301	0	0.0	1	1.3	0.286	5.75	0.23–142.80
0301/0302	0	0.0	2	2.6	0.144	9.71	0.46–204.7
0301/0303	1	0.7	0	0	0.775	0.63	0.03–15.56
0301/0501	1	0.7	1	1.3	0.644	1.91	0.12–30.94
0401/0301	0	0.0	2	2.6	0.144	9.71	0.46–204.7
0401/0302	0	0.0	1	1.3	0.286	5.75	0.23–142.80
0402/0302	3	2	1	1.3	0.686	0.63	0.06–6.14
0403/0302	9	6.1	0	0	0.104	0.09	0.01–1.63
0403/0402	2	1.4	0	0	0.526	0.37	0.02–7.87
**0701/0201**	**24**	**16.2**	**4**	**5.1**	**0.016**	**0.28**	**0.09–0.84**
0701/0303	2	1.4	1	1.3	0.965	0.95	0.09–10.62
0702/0201	0	0.0	1	1.3	0.286	5.75	0.23–142.80
0801/0301	3	2.0	0	0	0.382	0.26	0.01–5.19
1001/0201	0	0.0	1	1.3	0.286	5.75	0.23–142.80
1001/0302	2	1.4	0	0	0.526	0.37	0.02–7.87
1001/0501	6	4.1	3	3.8	0.939	0.95	0.23–3.90
1101/0301	18	12.2	14	17.9	0.236	1.60	0.74–3.38
1101/0302	1	0.7	2	2.6	0.238	3.87	0.35–43.35
1101/0501	0	0.0	2	2.6	0.144	9.71	0.46–204.7
1101/0602	1	0.7	0	0	0.775	0.63	0.03–15.56
1102/0301	7	4.7	6	7.7	0.363	1.68	0.54–5.18
1102/0302	0	0.0	1	1.3	0.286	5.75	0.23–142.80
1102/0602	1	0.7	0	0	0.775	0.63	0.03–15.56
1103/0201	0	0.0	1	1.3	0.286	5.75	0.23–142.80
1201/0301	1	0.7	2	2.6	0.238	3.87	0.35–43.35
1301/0501	3	2.0	0	0	0.382	0.26	0.01–5.19
1301/0603	10	6.8	0	0	0.089	0.08	0.01–1.45
1303/0301	5	3.4	0	0	0.227	0.17	0.01–3.05
1302/0302	0	0.0	1	1.3	0.286	5.75	0.23–142.80
1303/0302	2	1.4	0	0	0.526	0.37	0.02–7.87
1401/0501	5	3.4	0	0	0.227	0.17	0.01–3.05
1401/0602	3	2.0	0	0	0.382	0.26	0.01–5.19
1404/0503	1	0.7	0	0	0.775	0.63	0.03–15.56
1501/0501	1	0.7	0	0	0.775	0.63	0.03–15.56
**1501/0601**	**2**	**1.4**	**14**	**17.9**	**<0.001**	**15.97**	**3.53–72.32**
1501/0602	10	6.8	0	0	0.089	0.08	0.01–1.45
1502/0501	1	0.7	0	0	0.775	0.63	0.03–15.56
1601/0502	1	0.7	0	0	0.775	0.63	0.03–15.56

SLE: Systemic Lupus Erythematosus; *n*: number of individuals; (%): allele frequency; OR: odds ratio; CI: confidence interval; *p*: significance level (*p* value < 0.05).

## Data Availability

The data are available from the corresponding authors upon request.

## Supplementary Materials

The following supporting information can be downloaded at: https://www.mdpi.com/article/10.3390/jpm12121955/s1, Table S1: Frequency of DRB1 and DQB1 alleles among SLE patients without Nephritis and with Nephritis in Jordanians; Table S2: The frequency of HLA-DRB1/DQB1 haplotypes among SLE patients without LN and SLE patients with LN in Jordanians.

## References

[1] Xue K., Niu W.Q., Cui Y. (2018). Association of HLA-DR3 and HLA-DR15 Polymorphisms with Risk of Systemic Lupus Erythematosus. Chin. Med. J..

[2] Goulielmos G.N., Zervou M.I., Vazgiourakis V.M., Ghodke-Puranik Y., Garyfallos A., Niewold T.B. (2018). The Genetics and Molecular Pathogenesis of Systemic Lupus Erythematosus (SLE) in Populations of Different Ancestry. Gene.

[3] Barber M.R.W., Drenkard C., Falasinnu T., Hoi A., Mak A., Kow N.Y., Svenungsson E., Peterson J., Clarke A.E., Ramsey-Goldman R. (2021). Global Epidemiology of Systemic Lupus Erythematosus. Nat. Rev. Rheumatol..

[4] Fortuna G., Brennan M.T. (2013). Systemic Lupus Erythematosus. Epidemiology, Pathophysiology, Manifestations, and Management. Dent. Clin. N. Am..

[5] Buniello A., Macarthur J.A.L., Cerezo M., Harris L.W., Hayhurst J., Malangone C., McMahon A., Morales J., Mountjoy E., Sollis E. (2019). The NHGRI-EBI GWAS Catalog of Published Genome-Wide Association Studies, Targeted Arrays and Summary Statistics 2019. Nucleic Acids Res..

[6] Kwon Y., Chun S., Kim K., Mak A. (2019). An Update on the Genetics of Systemic Lupus Erythematosus. Cells.

[7] Morris D.L., Sheng Y., Zhang Y., Wang Y.F., Zhu Z., Tombleson P., Chen L., Cunninghame Graham D.S., Bentham J., Roberts A.L. (2016). Genome-Wide Association Meta-Analysis in Chinese and European Individuals Identifies Ten New Loci Associated with Systemic Lupus Erythematosus. Nat. Genet..

[8] Graham R.R., Ortmann W.A., Langefeld C.D., Jawaheer D., Selby S.A., Rodine P.R., Baechler E.C., Rohlf K.E., Shark K.B., Espe K.J. (2002). Visualizing Human Leukocyte Antigen Class II Risk Haplotypes in Human Systemic Lupus Erythematosus. Am. J. Hum. Genet..

[9] Barcellos L.F., May S.L., Ramsay P.P., Quach H.L., Lane J.A., Nititham J., Noble J.A., Taylor K.E., Quach D.L., Chung S.A. (2009). High-Density SNP Screening of the Major Histocompatibility Complex in Systemic Lupus Erythematosus Demonstrates Strong Evidence for Independent Susceptibility Regions. PLoS Genet..

[10] Cusick M.F., Libbey J.E., Fujinami R.S. (2012). Molecular Mimicry as a Mechanism of Autoimmune Disease. Clin. Rev. Allergy Immunol..

[11] Kim K., Bang S.Y., Lee H.S., Okada Y., Han B., Saw W.Y., Teo Y.Y., Bae S.C. (2014). The HLA-DRβ1 Amino Acid Positions 11-13-26 Explain the Majority of SLE-MHC Associations. Nat. Commun..

[12] Molineros J.E., Looger L.L., Kim K., Okada Y., Terao C., Sun C., Zhou X.J., Raj P., Kochi Y., Suzuki A. (2019). Amino Acid Signatures of HLA Class-I and II Molecules Are Strongly Associated with SLE Susceptibility and Autoantibody Production in Eastern Asians. PLoS Genet..

[13] Morris D.L., Taylor K.E., Fernando M.M., Nititham J., Alarcón-Riquelme M.E., Barcellos L.F., Behrens T.W., Cotsapas C., Gaffney P.M., Graham R.R. (2012). Unraveling Multiple MHC Gene Associations with Systemic Lupus Erythematosus: Model Choice Indicates a Role for HLA Alleles and Non-HLA Genes in Europeans. Am. J. Hum. Genet..

[14] Bentham J., Morris D.L., Cunninghame Graham D.S., Pinder C.L., Tombleson P., Behrens T.W., Martín J., Fairfax B.P., Knight J.C., Chen L. (2015). Genetic Association Analyses Implicate Aberrant Regulation of Innate and Adaptive Immunity Genes in the Pathogenesis of Systemic Lupus Erythematosus. Nat. Genet..

[15] Shimane K., Kochi Y., Suzuki A., Okada Y., Ishii T., Horita T., Saito K., Okamoto A., Nishimoto N., Myouzen K. (2013). An Association Analysis of HLA-DRB1 with Systemic Lupus Erythematosus and Rheumatoid Arthritis in a Japanese Population: Effects of *09:01 Allele on Disease Phenotypes. Rheumatology.

[16] Sun C., Molineros J.E., Looger L.L., Zhou X., Okada Y., Ma J., Qi Y., Kim-Howard X., Bhattarai K., Adler A. (2016). High-Density Genotyping of Immune-Related Loci Identifies New SLE Risk Variants in Individuals with Asian Ancestry. Nat. Genet..

[17] Zhang J., Zhan W., Yang B., Tian A., Chen L., Liao Y., Wu Y., Cai B., Wang L. (2017). Genetic Polymorphisms of Rs3077 and Rs9277535 in HLA-DP Associated with Systemic Lupus Erythematosus in a Chinese Population. Sci. Rep..

[18] Hachicha H., Kammoun A., Mahfoudh N., Marzouk S., Feki S., Fakhfakh R., Fourati H., Haddouk S., Frikha F., Gaddour L. (2018). Human Leukocyte Antigens-DRB1*03 Is Associated with Systemic Lupus Erythematosus and Anti-SSB Production in South Tunisia. Int. J. Health Sci..

[19] Mokbel A.N., Al-Zifzaf D.S., ElSawy W.S., ElGabarty S. (2015). Association of HLA-DQB1*06 with Susceptibility to Systemic Lupus Erythematosus in Egyptians. Egypt. Rheumatol..

[20] Langefeld C.D., Ainsworth H.C., Graham DS C., Kelly J.A., Comeau M.E., Marion M.C., Howard T.D., Ramos P.S., Croker J.A., Morris D.L. (2017). Transancestral Mapping and Genetic Load in Systemic Lupus Erythematosus. Nat. Commun..

[21] Aringer M., Costenbader K., Daikh D., Brinks R., Mosca M., Ramsey-Goldman R., Smolen J.S., Wofsy D., Boumpas D.T., Kamen D.L. (2019). 2019 European League Against Rheumatism/American College of Rheumatology Classification Criteria for Systemic Lupus Erythematosus. Ann. Rheum. Dis..

[22] Jarrar Y.B., Ghishan M. (2019). The Nudix Hydrolase 15 (NUDT15) Gene Variants among Jordanian Arab Population. Asian Pac. J. Cancer Prev..

[23] Khdair S.I., Jarrar Y.B., Jarrar W. (2021). Immunogenetic Prediction of VDR Gene SNPs: Lack of Association with Susceptibility to Type 1 Diabetes in Jordanian Patients. Diabetes Metab. Syndr. Obes. Targets Ther..

[24] Weber F., Knapp G., Ickstadt K., Kundt G., Glass Ä. (2020). Zero-Cell Corrections in Random-Effects Meta-Analyses. Res. Synth. Methods.

[25] Ruxton G.D., Neuhäuser M. (2013). Review of Alternative Approaches to Calculation of a Confidence Interval for the Odds Ratio of a 2 × 2 Contingency Table. Methods Ecol. Evol..

[26] MedCalc Software Ltd. Odds Ratio Calculator. https://www.medcalc.org/calc/odds_ratio.php.

[27] Schoonjans F., Zalata A., Depuydt C.E., Comhaire F.H. (1995). MedCalc: A New Computer Program for Medical Statistics. Comput. Methods Programs Biomed..

[28] Ohyama K., Sugiura M. (2018). Evaluation of the Association between Topical Prostaglandin F2α Analogs and Asthma Using the Jader Database: Comparison with β-Blockers. Yakugaku Zasshi.

[29] Kawasaki A., Hasebe N., Hidaka M., Hirano F., Sada K.E., Kobayashi S., Yamada H., Furukawa H., Yamagata K., Sumida T. (2016). Protective Role of HLA-DRB1∗13:02 against Microscopic Polyangiitis and MPO-ANCA-Positive Vasculitides in a Japanese Population: A Case-Control Study. PLoS ONE.

[30] Adwan M. (2018). Clinical and Serologic Characteristics of Systemic Lupus Erythematosus in the Arab World: A Pooled Analysis of 3,273 Patients. Arch. Rheumatol..

[31] Graham R.R., Ortmann W., Rodine P., Espe K., Langefeld C., Lange E., Williams A., Beck S., Kyogoku C., Moser K. (2007). Specific Combinations of HLA-DR2 and DR3 Class II Haplotypes Contribute Graded Risk for Disease Susceptibility and Autoantibodies in Human SLE. Eur. J. Hum. Genet..

[32] Castaño-Rodríguez N., Diaz-Gallo L.M., Pineda-Tamayo R., Rojas-Villarraga A., Anaya J.M. (2008). Meta-Analysis of HLA-DRB1 and HLA-DQB1 Polymorphisms in Latin American Patients with Systemic Lupus Erythematosus. Autoimmun. Rev..

[33] Ayed K., Gorgi Y., Ayed-Jendoubi S., Bardi R. (2004). The Involvement of HLA-DRBI*, DQA1*, DQB1* and Complement C4A Loci in Diagnosing Systemic Lupus Erythermatosus among Tunisians. Ann. Saudi Med..

[34] Pan C.F., Wu C.J., Chen H.H., Dang C.W., Chang F.M., Liu H.F., Chu C.C., Lin M., Lee Y.J. (2009). Molecular Analysis of HLA-DRB1 Allelic Associations with Systemic Lupus Erythematous and Lupus Nephritis in Taiwan. Lupus.

[35] Marchini M., Antonioli R., Lleò A., Barili M., Caronni M., Origgi L., Vanoli M., Scorza R. (2003). HLA Class II Antigens Associated with Lupus Nephritis in Italian SLE Patients. Hum. Immunol..

[36] Vasconcelos C., Carvalho C., Leal B., Pereira C., Bettencourt A., Costa P.P., Marinho A., Barbosa P., Almeida I., Farinha F. (2009). HLA in Portuguese Systemic Lupus Erythematosus Patients and Their Relation to Clinical Features. Ann. N. Y. Acad. Sci..

[37] Fernando MM A., Stevens C.R., Sabeti P.C., Walsh E.C., McWhinnie AJ M., Shah A., Green T., Rioux J.D., Vyse T.J. (2007). Identification of Two Independent Risk Factors for Lupus within the MHC in United Kingdom Families. PLoS Genet..

[38] Hussain N., Jaffery G., Sabri A.N., Hasnain S. (2011). HLA Association in SLE Patients from Lahore-Pakistan. Bosn. J. Basic Med. Sci..

[39] Farivar S., Tezerjani M.D., Shiari R. (2015). Association of HLA-DRB1 Alleles with Juvenile-Onset Systemic Lupus Erythematosus (SLE) in Iranian Children. Int. J. Pediatr..

[40] Dedhia L., Pradhan V., Ghosh K., Nadkar M., Parekh S. (2018). Association of Human Leucocyte Antigen (HLA) Class II with Systemic Lupus Erythematosis (SLE) Patients from Western India. Meta Gene.

[41] Rezaieyazdi Z., Tavakkol-Afshari J., Esmaili E., Orouji E., Pezeshkpour F., Khodadoost M., Mazhani M., Sandooghi M. (2008). Association of HLA-DQB1 Allelic Sequence Variation with Susceptibility to Systemic Lupus Erythematosus. Iran. J. Allergy Asthma Immunol..

[42] Kapitany A., Tarr T., Gyetvai A., Szodoray P., Tumpek J., Poor G., Szegedi G., Sipka S., Kiss E. (2009). Human Leukocyte Antigen-DRB1 and -DQB1 Genotyping in Lupus Patients with and without Antiphospholipid Syndrome. Ann. N. Y. Acad. Sci..

[43] Bolin K., Sandling J.K., Zickert A., Jönsen A., Sjöwall C., Svenungsson E., Bengtsson A.A., Eloranta M.L., Rönnblom L., Syvänen A.C. (2013). Association of STAT4 Polymorphism with Severe Renal Insufficiency in Lupus Nephritis. PLoS ONE.

[44] Gunawan A., Handono K., Kalim H., Susianti H., Eko M.H., Lawrence G.S., Hasanah D., Mertianti E., Candradikusuma D. (2018). The Importance of HLA-DRB1 Alleles in Patients with Lupus Nephritis. Turkish J. Immunol..

[45] Bhallil O., Ibrahimi A., Ouadghiri S., Ouzeddoun N., Benseffaj N., Bayahia R., Essakalli M. (2017). HLA Class II with Lupus Nephritis in Moroccan Patients. Immunol. Investig..

[46] Wadi W., Elhefny N.E.A.M., Mahgoub E.H., Almogren A., Hamam K.D., Al-Hamed H.A., Gasim G.I. (2014). Relation between HLA Typing and Clinical Presentations in Systemic Lupus Erythematosus Patients in Al-Qassim Region, Saudi Arabia. Int. J. Health Sci..

[47] de Holanda M.I., Klumb E., Imada A., Lima L.A., Alcântara I., Gregório F., Christiani L.F., Martins C.O., Timoner B.E., Motta J. (2018). The Prevalence of HLA Alleles in a Lupus Nephritis Population. Transpl. Immunol..

[48] Niu Z., Zhang P., Tong Y. (2015). Value of HLA-DR Genotype in Systemic Lupus Erythematosus and Lupus Nephritis: A Meta-Analysis. Int. J. Rheum. Dis..

[49] Xu R., Li Q., Liu R., Shen J., Li M., Zhao M., Wang M., Liao Q., Mao H., Li Z. (2017). Association Analysis of the MHC in Lupus Nephritis. J. Am. Soc. Nephrol..

[50] Tsokos G.C. (2011). Systemic Lupus Erythematosus. N. Engl. J. Med..

