# Supplementary material for: Immunogenetic Profiling of SLE and LN among Jordanian Patients
Source: J Pers Med. 2022 Nov 25;12(12):1955. doi: 10.3390/jpm12121955 (PMC9782219; doi:10.3390/jpm12121955)
Supplement: Supplementary file 1 [file jpm-12-01955-s001.zip › jpm-1991880-supplementary.pdf]

Table S1. Frequency of DRB1 and DQB1 alleles among SLE patients without Nephritis and with Nephritis in Jordanians

	SLE without Nephritis		SLE with Nephritis				
**DRB1**	**N=82**	**(%)**	**N=78**	**(%)**	***p* Value**	**OR**	**95% CI**
*0101	2	2.4	0	0	0.309	0.21	0.01-4.34
*0301	15	18.3	17	21.8	0.580	1.25	0.58-2.71
*0302	3	3.7	0	0	0.204	0.15	0.01-2.85
*0401	2	2.4	3	3.8	0.609	1.60	0.26-9.84
*0402	0	0	1	1.3	0.479	3.19	0.13-79.58
*0405	1	1.2	0	0	0.518	0.35	0.01-8.62
*0701	5	6.1	6	7.7	0.690	1.28	0.38-4.39
*1001	9	11	4	5.1	0.176	0.44	0.13-1.49
*1101	17	20.7	19	24.4	0.583	1.23	0.59-2.59
*1102	13	15.9	8	10.3	0.295	0.61	0.24-1.56
*1103	0	0	1	1.3	0.479	3.19	0.13-79.58
*1201	2	2.4	2	2.6	0.960	1.05	0.15-7.66
*1301	3	3.7	1	1.3	0.336	0.34	0.04-3.36
*1302	0	0	1	1.3	0.479	3.19	0.13-79.58
*1303	1	1.2	1	1.3	0.972	1.05	0.07- 17.12
*1402	2	2.4	0	0	0.309	0.21	0.01-4.34
***1501**	**6**	**7.3**	**14**	**17.9**	**0.042**	**2.77**	**1.01-7.63**
*1601	1	1.2	0	0	0.518	0.35	0.01-8.62
**DQB1**	**N**	**(%)**	**N**	**(%)**	***p* Value**	**OR**	**95% CI**
*0201	14	17.1	21	26.9	0.132	1.79	0.84-3.84
*0301	29	35.4	26	33.3	0.787	0.91	0.48-1.76
*0302	10	12.2	8	10.3	0.698	0.82	0.31-2.21
*0303	1	1.2	1	1.3	0.972	1.05	0.07-17.12
*0401	2	2.4	0	0	0.309	0.21	0.01-4.34
*0402	2	2.4	0	0	0.309	0.21	0.01-4.34
*0501	11	13.4	6	7.7	0.240	0.54	0.19-1.54
*0502	1	1.2	0	0	0.518	0.35	0.01-8.62
***0601**	**5**	**6.1**	**16**	**20.5**	**0.007**	**3.97**	**1.38-11.45**
*0602	7	8.5	0	0	0.062	0.06	0.01-1.14

SLE: Systemic Lupus Erythematosus; LN: Lupus Nephritis; n: number of individuals; (%): Allele Frequency; OR: odds ratio; CI: confidence interval; p: significance level. (P value <0.05).

Table S2. The frequency of HLA-DRB1/DQB1 haplotypes among SLE patients without LN and SLE patients with LN in Jordanians

	SLE without Nephritis		SLE with Nephritis				
**Allele**	**N=82**	**(%)**	**N=78**	**(%)**	***p* value**	**OR**	**95% CI**
0101/0201	2	2.4	0	0	0.309	0.21	0.01 – 4.34
0301/0201	11	13.4	13	16.7	0.565	1.29	0.54 - 3.08
0301/0301	0	0	1	1.3	0.479	3.19	0.13 – 79.58
0301/0302	2	2.4	2	2.6	0.960	1.05	0.15 - 7.66
0301/0303	1	1.2	0	0	0.518	0.35	0.01 – 8.62
0301/0501	0	0	1	1.3	0.479	3.19	0.13 – 79.58

0301/0602	1	1.2	0	0	0.518	0.35	0.01 – 8.62
0302/0402	3	3.7	0	0	0.204	0.15	0.01 – 2.85
0401/0301	0	0	2	2.6	0.279	5.39	0.25 – 114.12
0401/0302	2	2.4	1	1.3	0.590	0.52	0.05 – 5.85
0402/0302	0	0	1	1.3	0.479	3.19	0.13 – 79.58
0405/0401	1	1.2	0	0	0.518	0.35	0.01 – 8.62
0701/0201	3	3.7	4	5.1	0.650	1.42	0.31 – 6.58
0701/0301	2	2.4	0	0	0.309	0.21	0.01 – 4.34
0701/0303	0	0	1	1.3	0.479	3.19	0.13 – 79.58
0702/0201	0	0	1	1.3	0.479	3.19	0.13 – 79.58
1001/0201	0	0	1	1.3	0.479	3.19	0.13 – 79.58
1001/0302	1	1.2	0	0	0.518	0.35	0.01 – 8.62
1001/0501	7	8.5	3	3.8	0.221	0.43	0.11 – 1.72
1001/0602	1	1.2	0	0	0.518	0.35	0.01 – 8.62
1101/0301	12	14.6	14	17.9	0.570	1.28	0.55 – 2.96
1101/0302	3	3.7	2	2.6	0.691	0.69	0.11 – 4.26
1101/0501	2	2.4	2	2.6	0.960	1.05	0.15 – 7.66
1102/0301	11	13.4	6	7.7	0.240	0.54	0.19 – 1.53
1102/0302	0	0	1	1.3	0.479	3.19	0.13 – 79.58
1102/0601	0	0	2	2.6	0.279	5.39	0.25 – 114.12
1102/0602	2	2.4	0	0	0.309	0.21	0.01 – 4.34
1103/0201	0	0	1	1.3	0.479	3.19	0.13 – 79.58
1201/0301	2	2.4	2	2.6	0.960	1.05	0.15 – 7.66
1301/0301	0	0	1	1.3	0.479	3.19	0.13 – 79.58
1301/0302	1	1.2	0	0	0.518	0.35	0.01 – 8.62
1301/0602	2	2.4	0	0	0.309	0.21	0.01 – 4.34
1302/0302	0	0	1	1.3	0.479	3.19	0.13 – 79.58
1303/0201	0	0	1	1.3	0.479	3.19	0.13 – 79.58
1303/0302	1	1.2	0	0	0.518	0.35	0.01 – 8.62
1402/0301	2	2.4	0	0	0.309	0.21	0.01 – 4.34
1501/0201	1	1.2	0	0	0.518	0.35	0.01 – 8.62
**1501/0601**	**5**	**6.1**	**14**	**17.9**	**0.021**	**3.37**	**1.15 – 9.86**
1601/0502	1	1.2	0	0	0.518	0.35	0.01 – 8.62

SLE: Systemic Lupus Erythematosus; LN: Lupus Nephritis; n: number of individuals; (%): Allele Frequency; OR: odds ratio; CI: confidence interval; p: significance level. (P value <0.05).
